# Hybrid Carrageenans Versus Kappa–Iota-Carrageenan Blends: A Comparative Study of Hydrogel Elastic Properties

**DOI:** 10.3390/gels11030157

**Published:** 2025-02-22

**Authors:** Maria Alice Freitas Monteiro, Bruno Faria, Izabel Cristina Freitas Moraes, Loic Hilliou

**Affiliations:** 1 Institute for Polymers and Composites, University of Minho, 5800-048 Guimarães, Portugal; mariaamonteiro13@gmail.com (M.A.F.M.); bruno.faria@dep.uminho.pt (B.F.); 2 Department of Food Engineering, Faculty of Animal Science and Food Engineering (FZEA), University of São Paulo (USP), Postgraduate Programme in Materials Science and Engineering, Pirassununga 13635-900, SP, Brazil; bel@usp.br

**Keywords:** carrageenan, hydrogel, molecular mass, shear storage modulus

## Abstract

A comparison between the gel properties of blends of kappa- and iota-carrageenans (K+Is) and hybrid carrageenans (KIs) with equivalent chemical compositions is here presented. The objective is to assess under which conditions hybrid carrageenans are valuable alternative to blends of kappa- and iota-carrageenans for gelling applications and to contribute to the identification of phase-separated structures or co-aggregated helices. Phase states constructed in sodium chloride and in potassium chloride confirm that KIs build gels under a much narrower range of ionic strength and polysaccharide concentration. Hybrid carrageenans displayed salt specificity, forming gels in KCl but not in NaCl, highlighting their limited gelling potential in Na^+^ environments. A two-step gelation mechanism was found in both systems at lower ionic strengths and when iota carrageenan is the major component. The shear elastic moduli of KI gels are overall smaller than those of blends, but the opposite is observed at lower ionic strengths in KCl and in systems richer in iota-carrageenans. The nonlinear elastic properties of gels do not relate to the use of blends or hybrid carrageenans for their formulation. Instead, larger contents in iota-carrageenans lead to gels able to sustain larger strains before yielding to a fluid state. However, these gels are more prone to strain softening, whereas strain hardening is measured in gels containing more kappa-carrageenan, irrespective of their blend or hybrid structure.

## 1. Introduction

Carrageenans are a family of sulfated polysaccharides, extracted from red seaweeds, which have long been integral to the food industry due to their unique gelling, thickening, and stabilizing properties [1]. They play a crucial role in the formulation of a wide range of products, including dairy goods, meat products, and plant-based alternatives [1,2]. Carrageenan is currently the leading seaweed-derived food hydrocolloid and accounts for the sixth-largest share of the global hydrocolloid market (in terms of value), after guar gum, gelatin, xanthan gum, cellulose gum, and arabic gum [3]. The global carrageenan market was worth USD 850 million in 2022 and is expected to expand at an average yearly growth rate of 6.2%, reaching USD 1.55 billion by 2032 [4].

The focus of most commercial applications has been on the use of kappa-carrageenans and iota-carrageenans, either in pure forms or as blends, to achieve desired textural properties [5,6,7]. Extensive research has been conducted on these carrageenans, particularly in terms of their chemical structure, gelling mechanisms, and interactions with other food ingredients [8]. However, the increasing demand for kappa- and iota-carrageenans in both food and non-food industries [9] has recently exerted significant pressure on carrageenan production and seaweed farming [1], making it urgent to look for underexploited and sustainable resources.

**Figure 1:**
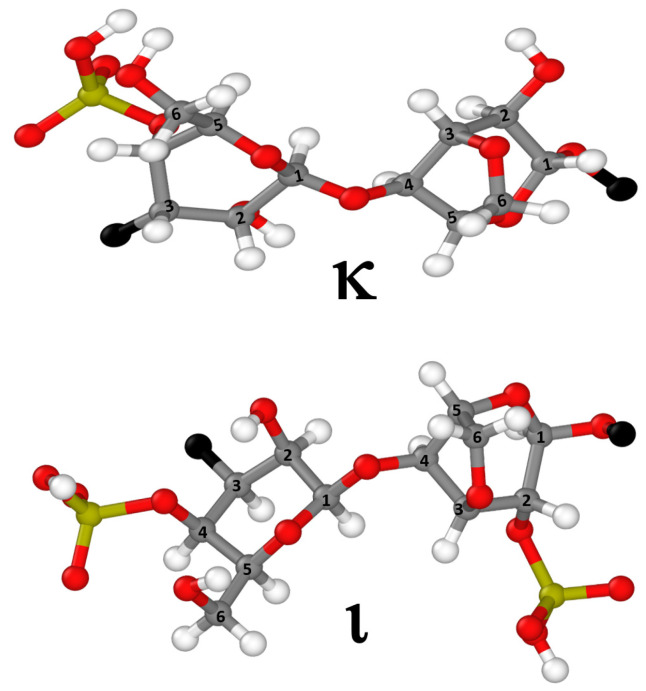
Chemical structures of the kappa-carrageenan diad (κ) and the iota-carrageenan diad (ι) arranged in sequences in the KI block copolymers or making up the whole polysaccharide chain in the corresponding homopolymers.

In this context, kappa/iota-hybrid carrageenans (KIs) [10], which consist of a random block copolymer of sequences of kappa-carrageenan (κ) and iota-carrageenan (ι) diads (disaccharides of D-galactose and 3,6-anhydrogalactose units linked by specific glycosidic bonds, see Figure 1), have emerged as promising alternatives to traditional commercial blends. Industrially known as weak kappa or Kappa-2 [11,12], these copolymers form thermo-reversible hydrogels with intermediate elasticity in comparison to those of kappa- and iota-carrageenan, offering unique structural and performance advantages in certain applications [11,12]. Moreover, they are predominantly sourced from wild seaweeds in cold waters, unlike kappa- and iota-carrageenans, which are mainly obtained from the intense farming of warm-water species, with associated production issues [13]. The ratio between kappa-carrageenan and iota-carrageenan diads in a KI is set by biology, that is, the family and genus of seaweeds used to extract the KI. So, instead of mixing at different ratios to tune the properties of the blend, the properties of a KI are tuned by choosing the correct seaweed and isolating the corresponding copolymer [10,14]. This makes hybrid carrageenans not only an attractive option for alternative sourcing but also a potentially more cost-effective solution by eliminating the need for blending different types of carrageenans.

Despite their promise, these copolymers present several ongoing research challenges. Their chemical structure is influenced by various factors, such as the species of algae, the time and place of harvest, and the parameters and methods used during extraction [14]. These factors do impact KI functionality, as much as they control the gelling properties of commercial kappa- and iota-carrageenans [15]. While these variables affect the mechanical and structural characteristics of the resulting hydrogels, the relationship between the gelation mechanism, the gel structure, and the chemical structure of hybrid carrageenans remains complex and poorly understood [10]. Additionally, while the gelling properties of commercial kappa- and iota- carrageenans are relatively well-documented, their gel structure–elastic properties relationships are far from being fully clarified [16].

Naturally, this is also the case for blends of kappa- and iota-carrageenans (here labeled as “K+I”) for which controversial models are proposed (see, e.g., [17] and references therein). By comparing the experimental results for K+I gel elasticity with blending rules, phase-separated networks [18] signaled by a two-step gelation [19,20,21], interpenetrated networks [22], and co-aggregated networks where helices of kappa- and iota-carrageenans are at least partially self-assembling [23] or form an interactive and associative network [24] have been proposed. The block copolymer nature of hybrid carrageenans impedes the phase separation of kappa- and iota-carrageenan blocks in the helical or coil conformation. Therefore, a systematic comparison of the solution and gel phases of KI and K+I appears as an elegant route to the elucidation of the blend microstructure in the gel phase. Interestingly, this route has not been much explored in the literature, though such a comparison turned out to be a strong argument in favor of the copolymer nature of KI by taking advantage of the ion specificity of kappa-carrageenan in the presence of potassium cations [25]. Indeed, kappa-carrageenan can be separated from iota-carrageenan in K+I systems prepared in potassium chloride, whereas KIs do not show any phase separation [25]. KI and K+I comparative phase states in both potassium chloride and sodium chloride were produced for a range of compositions varying between 47 mol.% and 78 mol.% of κ [26]. These studies were rather application-oriented as the main objective was to screen for regimes of polysaccharide concentration and ionic strength where KIs could be valuable alternatives to K+I gels.

Here, the range of blends and copolymer compositions is extended beyond the balanced 50% usually focused on in the literature [18,19,20,21,22,23,24,25], to nearly pure kappa- and iota-carrageenans. Following a recent study where a set of hybrid carrageenans were extracted from commercial seaweeds [27], these copolymers are here alkali-treated to produce KI with virtually no diads of the mu-carrageenan (μ) or nu-carrageenan (ν) types. These diads are more sulphated than κ and ν diads and are known to limit or impede gel formation [10]. The large amplitude oscillatory shear (LAOS) behavior of produced KI and K+I gels is reported as it has received less attention in the carrageenan literature [10,16,28]. Although LAOS is less frequently used nowadays due to the lack of a unified interpretation of the results [29], it has given interesting structural characterization when the results are fitted with filament network theories or colloidal gel theory for strain-hardening gels (see, e.g., [16,30,31,32]). LAOS characteristics are expected to complement the structural information inferred from the gels’ mechanical spectra measured for various polysaccharide and salt concentrations. Overall, the approach set here is expected to contribute to the identification of different gel structures in KI and K+I but also to define the conditions under which hybrid carrageenans can be most effectively used to replace iota- and kappa-carrageenan blends, in particular during industrial processing where large deformations are at play.

## 2. Results and Discussion

### 2.1. Characterization of Produced Carrageenans

The chemical structures of all extracted and alkali-treated carrageenans are displayed in Table 1. The proton NMR spectra used for the quantitative estimation of the carrageenan diads can be found in Figures S1 and S2 in the Supplementary Materials. All extracted polysaccharides are copolymers, whereas the commercial kappa-carrageenan is a homopolymer since only κ could be detected in the respective NMR spectrum. All samples have no or negligible amounts of μ and ν (given the limit of resolution of proton NMR), with the exception of samples F and B′. While the comparison between samples E and F allows for assessing the impact of few ν diads on the phase state, sample D′ was extracted from another seaweed to replace sample B′, since D′ has the same ι content but with virtually no ν. A certain amount of Floridean starch (circa 10 mol.% of total polysaccharides) was found in samples F, E′, and F′. Therefore, a new KI, sample H′, was produced and treated with NaOH to compare with sample E′ and thus assess the impact of Floridean starch.

The molecular mass distributions of all carrageenans are shown in Table 1. Interestingly, the type of alkali modification has a strong effect on both the weight-averaged molecular mass Mw and the width of the distribution (PDI) for samples C and C′ and samples K and K′. For these two pairs of samples, the modification by NaOH (samples C′ and K′) gives hybrid carrageenans with a larger Mw and very different PDI when compared with the polysaccharides treated with KOH (samples C and K). Strong alkali treatments are known to reduce the Mw of carrageenans [33]. Also, the endogenous salts brought by the dried seaweeds [34] may add to the alkali modification of the molecular mass distribution. We thus suspect that the seaweeds used to produce samples C and K are rich in potassium salt, thereby increasing the ionic strength of KOH and promoting a larger chain scission than with NaOH. In contrast to this, the remaining hybrid carrageenans show either similar molecular mass distributions (see samples B-B′, E-E′, and F-F′) or slightly different Mw (see samples I-I′, J-J′, and K-K′). Mw is known to be a critical parameter for gel formation in carrageenans. In particular, it has been recently shown that the critical mass Mc below which no gel can be formed in KCl depends on the chemical structure of the carrageenans [35]. Longer iota-carrageenan chains are needed to obtain a gel in 0.1 M KCl at a polysaccharide concentration of 1 wt.% when compared with KI chains. Note here that all Mw values reported in Figure 1 are above the Mc found for various hybrid carrageenans and non-commercial kappa- and iota-carrageenans [35].

### 2.2. Phase States in KCl and NaCl

**Figure 2:**
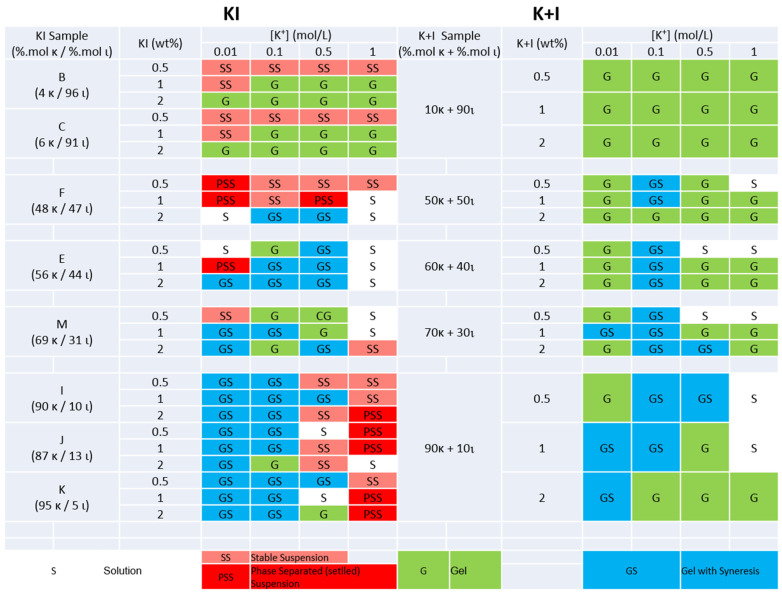
Phases formed in KCl by hybrid carrageenans (KI, left columns) and by carrageenan blends (K+I, right columns) with equivalent chemical composition in κ and ι, as a function of 2 factors: polysaccharide concentration (KI or K+I, in wt.%) and ionic strength of K^+^ (in mol/L).

**Figure 3:**
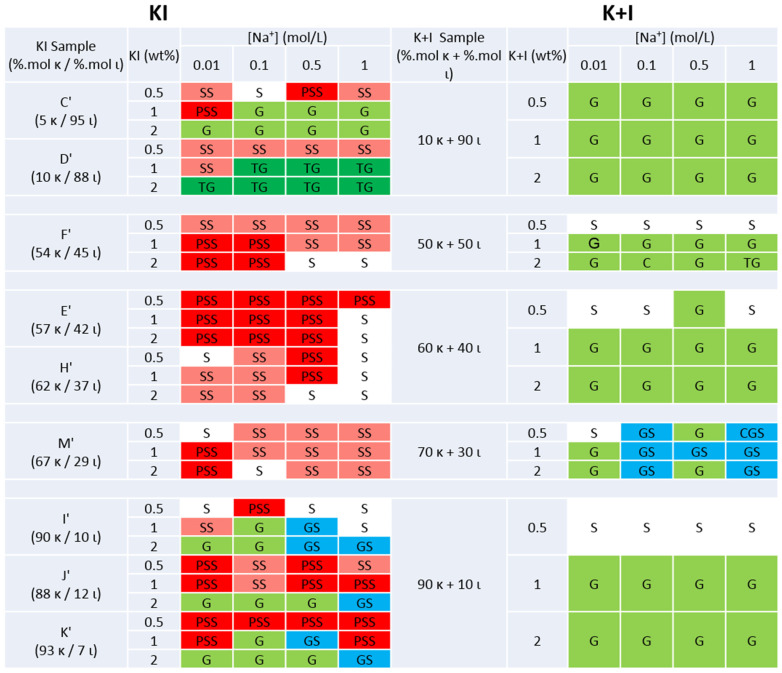
Phases formed in NaCl by hybrid carrageenans (KI, left columns) and by carrageenan blends (K+I, right columns) with equivalent chemical composition in κ and ι, as a function of 2 factors: polysaccharide concentration (KI or K+I, in wt.%) and ionic strength of Na^+^ (in mol/L). Phase labeling is as in Figure 2.

The gel or solution state of all hybrid carrageenans and blends of kappa- and iota-carrageenan prepared under various salt conditions and polysaccharide concentrations are presented in Figure 2 and Figure 3 for KCl and NaCl, respectively. Elemental analysis showed that, due to the alkali treatment, all produced carrageenans brought Na^+^ and K^+^ in amounts varying between 11 and 40 wt.%. Thus, at the largest carrageenan concentrations tested in Figure 2 and Figure 3 (2 wt.%), the actual ionic strengths are modified by 0.02 M for K^+^ and 0.03 M for Na^+^, at most. Representative images of the samples’ phases sorted into solutions, suspensions (stable or settled), and gels (with or without water syneresis) are shown in Figure S3 in the Supplementary Materials. Overall, the comparison of the phases in the right and left columns in Figure 2 and Figure 3 confirmed notable differences between KI and blends [12,26]. Note here that the phase states found for pure iota-carrageenan resemble the phases reported here for the blend with composition 10K+90I, with the exception of low salt conditions (0.01 M), where Michel et al. [36] reported the absence of gels. As for a comparison of pure kappa-carrageenan with the 90K+10I blend, the same authors found similar phases, except at 0.01 M KCl where pure kappa-carrageenan did not form gels at 0.5 and 1 wt.%, and also in NaCl where pure kappa-carrageenan only formed gels at 2 wt.% for ionic strengths larger than 0.1 M [36].

Gels (labeled in green in Figure 2 and Figure 3) in the presence of K^+^ occurred more readily in blends than in KI. For ι-rich samples (B and C), gelation was more dependent on the polysaccharide concentration, occurring only at copolymer concentrations ≥ 1 wt.%, indicating a threshold for forming a stable network. Below this concentration, KI samples formed stable suspensions of aggregates, suggesting that the latter are insufficient in number and size to form a fully connected network. Given the high charge density of ι, this behavior is expected under low K^+^ concentrations, where monovalent salts like KCl provide enough ionic shielding but not enough for stable gel network formation [8]. In contrast, all blends formed gels across similar conditions.

For κ-rich KI samples, ionic strength played a more significant role, with gel formation particularly challenged at elevated K^+^ concentrations (≥0.5 mol/L for samples J, and >1 mol/L for the corresponding blend). While potassium ions more effectively induce the helical and aggregated states of kappa-carrageenan, salt-out effects at large ionic strength may lead to precipitation, compromising gel formation. A similar high salt sensitivity was observed in samples E and M at a K^+^ concentration of 1 mol/L, where stable suspensions or solutions are observed, whereas low ionic strengths and low polysaccharide concentrations hindered the gelation.

Sample F showed difficulty in forming gels, which only occurred at high polysaccharide concentrations (2 wt% KI) and ionic strengths of 0.5–1 mol/L, in evident contrast to the blend with equivalent composition in kappa- and iota-carrageenan diads. The content of precursor units in sample F (≈5 mol.%, see Table 1) may explain such a discrepancy as the presence of ν and μ diads in the KI chains is known for their loss in gel ability [10,37], which is confirmed by the gelling ability of the precursor-free sample E showing similar Mw. Alternatively, the presence of Floridean starch in F could hinder the gel phase seen in the corresponding blend. The existence of a cut-off content in κ (circa 50 mol.%) needed for the gel formation of hybrid carrageenan under specific KCl and polysaccharide concentrations emerges from the differences between sample F and samples E and M, whose phases in KCl compare well with those of the blends and the phase state found for a hybrid carrageenan containing 53 mol.% κ [26]. The latter study reported that KI produced gels with no water syneresis, in contrast to gels made from the blends. The data in Figure 2 confirm the syneresis in blends, but samples E, F, and M are also prone to water expulsion from the network. This discrepancy suggests that possible differences in the Mw as well as in the distributions of κ and ι sequences along the KI chains, which are inherent to the different algal sources used here, give different gel syneresis. Note also that slight differences in the experimental protocols employed (in particular the thermal history during cooling) or in the assessment of water syneresis (subjected to experimentalists’ criterions) can also lead to such discrepancies.

In NaCl, the differences between KI and blends are even more striking, which points toward the Na^+^ specificity of these copolymers when they contain between 50 and 90 mol% κ. The gel formation for blends containing more kappa-carrageenan was mostly conditioned by low polysaccharide concentrations, not showing much dependence on the ionic strength (contrarily to what is seen in KCl). As expected, due to the non-specific ion response of iota-carrageenan [8], ι-rich KI samples (C′ and D′) demonstrated consistent gelling behaviors in both Na^+^ and K^+^. In contrast, κ-rich samples (I′, J′, and K′) required higher polysaccharide concentrations to form gels in NaCl than in KCl.

Notably, for samples F′, E′, H′, and M′ with 50–70 mol.% κ, no gels formed under any condition, contrasting starkly with the corresponding blends. The differences with samples E′ and F′ in Figure 3 suggest that the distributions of κ and ι blocks as well as their lengths are chemical variables influencing the overall gelling performance of KI in NaCl. The effect of Floridean starch on the gelling ability of KI in NaCl could not be assessed as samples H′ and E′ did not form gels.

Overall, as much as the gelling functionalities of carrageenans are at play, the results displayed in Figure 2 and Figure 3 show that hybrid carrageenans can be a valuable alternative to blends of kappa- and iota-carrageenan but only at specific salt and polysaccharide concentrations. Nonetheless, rheological testing is essential for a comparative estimation of gel elasticity and resistance to deformation.

### 2.3. Gel Formation During Cooling

Samples C, E, K, C′, and K′ (sample E′ did not form gels) were chosen for rheometry, as these differed notably in κ- and ι-carrageenan contents, enabling the investigation of rheological responses tied to the κ-content. This selection also facilitated the evaluation of how polysaccharide concentration and ionic strength influence rheology since these samples formed gels under a broader range of conditions, facilitating direct comparisons within each sample. Given the NMR-related uncertainty in the κ-carrageenan contents in samples C and C′, and because 10 wt.% kappa-carrageenan is expected to impact more than 5 wt.% on the gel rheology, a K+I sample with a composition 10K+90I was chosen for comparison with samples C and C′. KI from *M. stellatus*, which resembles samples M and M′, has been extensively studied under similar conditions [38,39,40]. Thus, the rheological characterization of sample E was preferred here. It gives a unique opportunity to explore less-documented carrageenan types, adding depth to the rheological findings, in particular in a composition of κ and ι close to 50 mol.%, which has been much studied in blends of kappa- and iota-carrageenan [17,18,19,20,21,22,23,24]. Similar concentrations and ionic strengths were chosen for all selected KI and K+I whenever possible. However, sample K and the corresponding blend did not gel or formed gels with significant water syneresis, precluding any rheological comparison. Thus, a different concentration or ionic strength was chosen.

**Figure 4:**
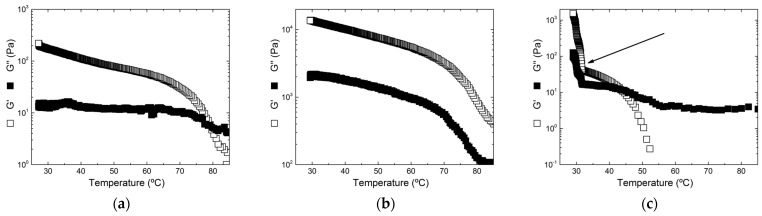
Temperature dependence of shear storage modulus G′ and loss modulus G″ during the cooling of (**a**) a blend 10% + 90% K+I at 1 wt.% in 1 M KCl, (**b**) sample E at 2 wt.% in 0.5 M KCl, and (**c**) sample C at 2 wt.% in 0.01 M KCl. The arrow in (**c**) indicates the temperature T2 where a second step starts during the gel formation.

Figure 4 gives illustrative thermorheological curves measured during the cooling of hot KI and K+I solutions in order to assess the sol–gel transitions. All cooling curves are available in Figures S4 and S5 in the Supplementary Materials, for gels formed in KCl and NaCl, respectively. Three types of cooling were observed. A clear sol-to-gel transition characterized by the crossover between the shear storage modulus G′ and the shear loss modulus G″, occurring at the gel transition temperature Tg, can be inferred from the data such as those plotted in Figure 4a,c.

A monotonic increase of both moduli with the decreasing temperature, where no crossover is detected and thus no Tg, is displayed in Figure 4b. This type of cooling suggests that gelation occurred at temperatures above 85 °C as G′ is larger than G″ in the whole temperature range tested. Note that for sample C′ at 2 wt.% in 1 M NaCl, G′ is always smaller than G″ during the whole cooling down to 25 °C (see Figure S5C). This indicates that sample C′ remained in the liquid state during cooling. The crossover between G′ and G″ occurred during the time spent at 25 °C for the record of the remaining gel properties (see Figure S9C showing G′ > G″ at large strains). Finally, the cooling illustrated in Figure 4c shows two steps in the thermal evolution of G′ (or G″). A temperature T2 (indicated by an arrow) lower than Tg signals the onset of a second gelling process corresponding to the elastic reinforcement (increase in G′) of the gel network established at Tg. Two-step gelation has been reported in K+I (see for instance [19,20,22,24,41]) and in hybrid carrageenan samples [42]. Tg and T2 are listed in Table 2 for all samples tested in KCl and in Table 3 for all samples tested in NaCl.

In KCl, both ι-rich hybrid carrageenan samples (sample C) and their corresponding commercial blends displayed two-step gelling mechanisms. Despite their high ι-content (≈90 mol.%), their gelling behavior deviated significantly from the monotonic increase in G′ and G″ typically observed in pure iota-carrageenan [23]. Because iota-carrageenan typically gels at a higher Tg than kappa-carrageenan, though the difference in Tg depends on the salt type and concentration [8], the presence of Tg and T2 suggests that even small κ fractions (≈10 mol.%) can form microdomains or secondary aggregates that reinforce the iota-carrageenan gel network and contribute to a second gelation step. Heterogeneous microdomains related to phase-separated or interpenetrated kappa- and iota-carrageenan assemblies have also been evidenced in K+I using scattering techniques [18,23], NMR [18,43], optical microscopy [23], and particle tracking [44].

Previous studies [20,22] have reported the formation of phase-separated and/or interpenetrating networks in blends, highlighting that κ fractions as low as 2.5% can significantly influence their rheological behavior. Additionally, residual κ-content in the commercial iota-carrageenan (see Table 1) likely amplifies its contribution to network formation in the 10K + 90I blend, raising the actual κ-content to ≈17.2 mol.%. This higher effective κ fraction may explain the formation of a secondary gelation phase in these ι-rich systems. However, since Tg and T2 also show up in hybrid carrageenans, a two-step gelation cannot be systematically associated with phase separation. At large compositions in kappa-carrageenan in the blend and κ-content in the hybrid carrageenan, gelling temperatures were only recorded at low salt and carrageenan concentrations, and a single thermal process (Tg) was mainly recorded. Either torque limitation at higher temperatures impedes a clear probe of the networking of the smaller ι fraction or the elasticity of the κ network, building up at temperatures closer to the ι network [8], masks the much softer ι one.

In NaCl, two-step gelation in ι-rich samples was only observed at low ionic strengths, transitioning to a single-step mechanism at 1 M NaCl. Under such ionic strength, gelling processes are better resolved in blends than in KI (see Figure S5). At 2 wt.%, sample C′ failed to gel within the defined temperature range despite displaying gel-like behavior (see Figure 3), which points toward the role of the cooling history on the resulting gel structure and elasticity. All κ-rich samples exhibited single-step gelation mechanisms. The absence of a clear two-step mechanism in κ-rich systems, opposed to what was observed in ι-rich ones, again reflects the elastic dominance of kappa-carrageenan and κ-block helices in the forming gel network. Furthermore, gelation onset appeared as a step rise in G′ nearly independent of ionic strength and occurred at relatively lower temperatures, between 40 °C and 55 °C, when compared with KCl. No crossover between G′ and G″ could be detected in all samples but sample K′ at 0.01 M NaCl.

### 2.4. Gel Elasticity and Structure

**Figure 5:**
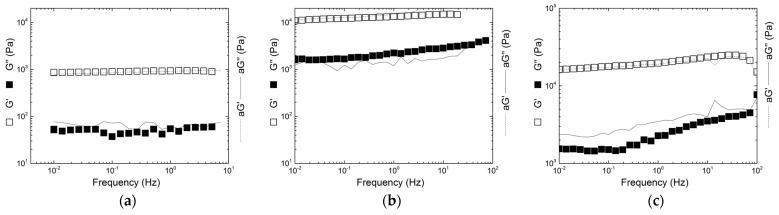
Mechanical spectra of KI gels (symbols) and corresponding K+I gels (lines for the moduli vertically shifted by a factor a): (**a**) sample C and K+I (10%K + 90%I) at 2 wt.% in 1 M KCl, (**b**) sample E and K+I (60%K + 40%I) at 2 wt.% in 0.5 M KCl, and (**c**) sample K and K+I (90%K + 10%I) at 2 wt.% in 0.5 M KCl.

The mechanical spectra of selected gels formed in KCl are reported in Figure 5, whereas all remaining spectra are reproduced in Figures S6 and S7 in the Supplementary Materials. Essentially, all copolymer gels and respective blend counterparts presented solid-like mechanical spectra, with G′ > G″, throughout all the considered range of frequency, and G′ being nearly frequency-independent. For all samples but three in KCl, it was possible to superimpose the spectra of KI gels on those of K+I formed under similar conditions by vertically shifting the G′ and G″ curves of KI gels. This is achieved by multiplying both moduli of KI gels by a shift factor a (see left vertical axes in Figure 5). This shift factor reflects the concentration dependence of the moduli in networks presenting structural self-similarity at all length scales for all concentrations considered [16]. Such vertical superimposition indicates that the elastic structure is overall similar in both KI and K+I gels. Differences in the concentration scaling of G′ and G″ are responsible for the different levels of viscoelasticity in KI and K+I gels, and horizontal shifting of the curves is thus not needed to achieve a superimposition. This is again in favor of some degree of co-aggregation of kappa- and iota-carrageenan helices in blends as no phase separation between a κ-rich network and a ι-rich network occurs in hybrid carrageenans due to the covalent bonds between the random κ and ι blocks. Note however that different network structures may generate identical mechanical spectra. Therefore, the data in Figure 5 are not a sufficient proof for helical co-aggregation in blends, and phase separation cannot be excluded. Superimposition failed in sample C at 0.01 M KCl due to the slow gelling kinetics occurring during the record of the spectra. For sample E with 2 wt.% in 0.01 M KCl, the local minimum in G″ is shifted to higher frequencies with respect to the blend sample, indicating faster relaxation processes in the hybrid carrageenan than in the blends which impede the superimposition of the two spectra.

In NaCl, the mechanical spectra of gels from KI and K+I could not be superimposed in any gelling conditions. This is due to very different levels of elastic moduli (see below with *G0*) and solid-like behavior (estimated from the gap between G′ and G″ values) originating from different gel structures. An exception though is found for the spectrum of sample K′ gelled in 0.5 M NaCl with 2 wt.% KI, which superimposed onto the spectrum shown by the corresponding blend. Taking on board the concept of structural self-similarity for all concentrations, which is implied by the power law dependence of the gel elasticity with the carrageenan concentration [16], the absence of superimposition confirms that overall the structure of hybrid carrageenan gels in NaCl differs from the structure of the blends, at least for the range of concentrations and ionic strength tested here.

**Figure 6:**
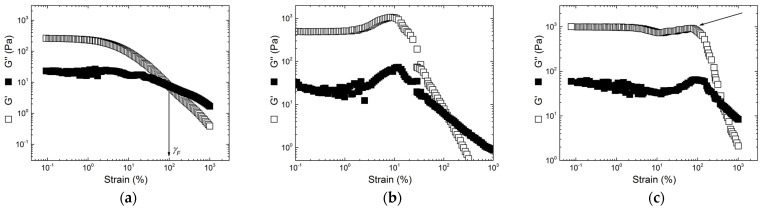
Strain sweeps performed on (**a**) sample K gelled in 0.5 M KCl with a concentration of 0.5 wt.%, (**b**) sample K gelled in 0.01 M KCl with a concentration of 0.5 wt.%, and (**c**) sample C gelled in 1 M KCl with a concentration of 2 wt.%. The arrow in (**a**) shows the strain *γ_F_* for the strain-induced fluidization of the gel. The arrow in (**c**) indicates the strain hardening showing up after the first strain softening at a smaller strain.

The gels’ rheological responses to strain sweeps from small to large amplitude oscillatory shear (LAOS) are presented in Figure 6 for selected gels; the remaining curves being available in Figures S8 and S9 in the Supplementary Materials, respectively, for gels formed in KCl and NaCl. At smaller strains, G′ and G″ are constant, allowing for the identification of the linear regime of viscoelasticity, from which the elastic modulus of the gels’ *G0* is inferred. Each *G0* is computed from the mean values of G′ averaged over one decade of strain in the linear regime. The *G0* values listed in Table 2 and Table 3 indicate that commercial blends generally produced more elastic gels than the corresponding hybrid carrageenans. There are few exceptions though, namely, the gel from sample E in 0.5 M KCl, which is more than 10-times more elastic than the gel made from blending 60% kappa-carrageenan with 40% iota-carrageenan, and gels from samples C and K′ at lower ionic strengths. These KIs are thus industrially attractive as they could replace the corresponding blends at a lower cost since less carrageenan will be needed to match their elastic performance. Increasing the κ-content in hybrid carrageenans or the kappa-carrageenan fraction in the blend leads to an increase in *G0* for all ionic strengths and carrageenan concentrations tested. This is in harmony with the wide amount of literature on the larger gel elasticity exhibited by kappa-carrageenan gels when compared with iota-carrageenan gels [19,20,22,24,36] and on the monotonic increase in *G0* with the κ fraction in KI [25,26,27,28,37]. As expected from previous studies on KI [16] and K+I [17,24], the increase in the polysaccharide concentration is accompanied by an increase in *G0*. However, an increase in ionic strength does not systematically lead to increased gel elasticity. This is the case for the copolymer gels rich in ι (samples C and C′) or κ (samples K and K′), whereas a single blend (60% kappa-carrageenan + 40% iota-carrageenan) showed a smaller gel elasticity at larger KCl concentrations. These results are overall in agreement with the existence of a maximum in *G0* as the ionic strength is increased for KI [26] and in K+I gels [23].

Under large deformation, when both moduli G′ and G″ are strain-dependent, gels exhibited strain-hardening or strain-softening behavior, as summarized in Table 2 and Table 3. Strain softening is depicted in Figure 6a and reflects a decrease in G′ with the increasing strain. Strain softening was measured for 17 gels out of a total of 30 and is typically associated with structural strain-induced orientation, rearrangement, or breakdown. The breakdown, or strain-induced fluidization, occurs at a critical strain *γ_F_* where G′ = G″, beyond which a fluid-like behavior shows up with G″ > G′. The strain-hardening behavior, characterized by an increase in both G′ and G″ with the shear strain up to a local maximum, is illustrated in Figure 6b. In many biological networks, strain hardening is explained by the stretching of semiflexible filaments pined on a three-dimensional network (see, e.g., [31,32,45] and references therein). Thus, the strand-like structure of carrageenan gels [8,16], modulated by the bending rigidity of the filaments and the topology of the network [31,32,45], can be called here to rationalize the increase in G′ as strain increases.

Notably, a third type of nonlinear behavior was measured with six gels and is illustrated in Figure 6c. After the linear regime, the moduli first drop before increasing at an intermediate strain, suggesting a local hardening with a maximum in G′ (signaled by an arrow in Figure 6c), and eventually drop to the strain-induced fluidization. This softening/hardening response (quoted as SOFT/HARD in Table 2 and Table 3) is more frequent in blends rich in iota-carrageenans, whereas strain hardening is essentially measured in gels richer in κ-content, being either blends or hybrid carrageenans (see Table 2 and Table 3).

Strain hardening has been recently found in KI gels [16,28,30] and gels made from blends of kappa-carrageenan and iota-carrageenan [18,28]. In particular in blends, the strain hardening was attributed to phase-separated aggregates of iota-carrageenan helices [18,28], since iota-carrageenan gels showed strain hardening in various gelling conditions [16,18,28]. The strain hardening reported in Table 3 for κ-rich KI and blends is thus hard to identify with the aggregates made of iota-carrageenan helices. Note here that gels were formed in the plates of the rheometer under a constant gap, since the thermal expansion of the shearing tools was compensated during cooling. In a previous study, gels were formed under a constant normal force condition [28], set to 0 N, with a view to adjusting the sample volume change during gel formation to the volume between the shearing plates. Clearly, the strain hardening needs to be further studied using gelling conditions controlled by normal stresses before concluding about its relationships with the carrageenan or blend composition. Indeed, a certain amount of pre-tension applied on the filaments during network formation is theoretically needed to produce a significant strain-hardening behavior [32].

The data in Table 2 and Table 3 show that gels with a smaller *G0* show a larger *γ_F_*, independent of the strain-hardening or -softening behavior. Practically, a larger *γ_F_* influences the gels’ resilience and functionality in real-world applications, as these gels present a higher rupture resistance under high-strain conditions. Larger *γ_F_* values were systematically found in gels made of blends having larger ι-contents, a property long known from food engineers [8]. The larger strain resistance of iota-carrageenan-rich blends has been more recently documented in a study where a phase inversion, and thus phase separation, was inferred from the dependence of *γ_F_* with the ι fraction [19]. The novelty here lies in the ι–rich KIs as good candidates to replace blends for applications where larger *γ_F_* values are needed, irrespective of hardening or softening attributes.

## 3. Conclusions

Starting with a set of hybrid carrageenans (KIs) extracted from commercial seaweeds, a subset was selected to conduct a comparative study with blends of kappa- and iota-carrageenans (K+Is) showing an equivalent composition in terms of contents in carrageenan diads (κ- and ι-content). The states constructed in various salt conditions for each class of gelling systems are different. Gel formation occurred more readily in blends than in hybrid carrageenans, across almost all chemical compositions, polysaccharide concentrations, and ionic strengths. In spite of using carrageenans with molecular masses larger than the critical masses Mc needed for gel formation at 1 wt.% in 0.1 M KCl, one may question whether the bigger propensity for blends to form gels under other gelling conditions relates to the fact that both kappa- and iota-carrageenans showed larger molecular masses than those of produced copolymers. Using samples with identical molecular masses will only partially address this shortcoming as the Mc for each gelling condition and type of carrageenan is to be quantified in the first place. Such Mc determination should deserve a dedicated study. Hybrid carrageenans demonstrated gelation behavior comparable to blends under specific conditions, such as higher polysaccharide concentrations for ι-rich samples and κ/ι-balanced samples in KCl and NaCl, and lower ionic strengths for κ-rich samples in KCl. KIs displayed salt-specificity, forming gels in KCl but not in NaCl, highlighting their limited gelling potential in Na^+^ environments. In terms of gel rheological properties, K+I generally produced stiffer gels than KI. However, under high ionic strengths, KI significantly outperformed the commercial blends in gel elasticity. Similarly, at low ionic strengths, certain KIs exhibited comparable or even superior elasticity. Increased elasticity often came at the expense of reduced resistance to deformation, leading to greater gel brittleness related to their larger κ-content. From a practical perspective, all the results reported in the present study underscore the importance of aligning the choice of substituting carrageenan blends by hybrid carrageenans with the specific functional requirements of the target application, although 1 M NaCl is clearly not relevant for food applications. This is due to the tradeoff between elasticity, strain, and salt resistance exhibited by both KI and K+I.

The results from the thermorheological study suggest some degree of co-aggregation between kappa-carrageenan helices and iota-carrageenan helices in the blends. Similarities between KI and K+I, such as two-step gelation during cooling, qualitatively identical gel mechanical spectra allowing the superimposition of data, and the coupling between the strain softening of brittle kappa-carrageenan aggregates and the strain hardening at a larger strain of iota-carrageenan aggregates, are in favor of this structural picture. Nevertheless, more study on the large deformation behavior is needed to rationalize the strain hardening by the filament structure of the carrageenan gels and to relate such a strand-like structure to the respective levels of co-aggregation and phase separation between iota- and kappa-carrageenan helices.

## 4. Materials and Methods

### 4.1. Materials

Red seaweeds producing hybrid carrageenans with various compositions in ι and κ diads were purchased from Algaplus (Ilhavo, Portugal) or were donated by Cargill Texturing Solutions—Hydrocolloids (Carentan, France). The selected seaweeds belong to the following genera: Euchema, Iridaea, Mastocarpus, and Kapphycus. Iota-carrageenan (lot SLCK5746) and kappa-carrageenan (lot BCCJ0930) are from Merck (Darmstadt, Germany). Potassium hydroxide, potassium chloride, sodium hydroxide, and sodium chloride (all from Sigma-Aldrich Química SL, Sintra, Portugal) were of a purity > 98%-. Ethanol (96 *v*/*v*%) was purchased in local supermarkets.

### 4.2. Carrageenan Extraction and Alkali Modification

Dried seaweed samples (1.5 g) were soaked in 50 mL of distilled water, at 80 °C for 3 h, with stirring every 30 min. After this initial extraction, 100 mL of distilled water was added to the algal suspension, which was then homogenized in a blender to enhance extraction efficiency. For samples I, J, and K, the protocol was adjusted by increasing the water volume to 150 mL to reduce the viscosity of the algal suspension and facilitate the solid–liquid separation. The extraction process was then continued at 90 °C for an additional hour. Following this, the solution was centrifuged at 8000 rpm for 10 min to recover the liquid phase, which was subsequently dried in an oven at 55 °C overnight to form carrageenan biofilms.

For the alkali modification, the films resulting from the carrageenan extraction were treated with 3 wt.% NaOH to produce carrageenans in the Na^+^ form or 3 wt.% KOH to produce carrageenans in the K^+^ form. Films (2 wt.%) were added to the corresponding alkaline solutions stirred at 85 °C during 3 h. An ethanolic precipitation (cold ethanol/carrageenan solution ratio of 2.5/1) was carried out to isolate the solid carrageenan by filtration with a metallic net. The resulting carrageenan was then dried at 55 °C overnight in an oven with air convection.

### 4.3. Carrageenan Characterization

Proton nuclear magnetic resonance (NMR) spectra of the extracted carrageenan were acquired using a Bruker Avance III spectrometer (Billerica, MA, USA) at 400 MHz. Carrageenan solutions (0.5 wt.% in D_2_O) were prepared and ultrasonicated with a DCG-300H bath (MCR Ltd., Holon, Israel) during several hours to lower the solution viscosity before NMR spectroscopy. The D_2_O peak was used as a reference signal after signal acquisition at 70 °C. NMR spectra were further referenced with respect to the peak assigned to κ (showing up at 5.09 ppm [46]) or to ι (showing up at 5.29 ppm [46]) and analyzed using NMRium^®^ online software (DEMO version). The molar fractions (mol.%) of the carrageenan diads (κ, ι, μ, and ν) were calculated based on the integrated intensity of the respective peaks divided by the sum of the integrated intensities of all peaks assigned to diads’ anomeric protons.

The molecular mass distribution was determined using size exclusion chromatography (SEC) (Waters 600 system with a Waters 2410 differential refractive index detector, Waters Portugal, Lisboa, Portugal). The system was equipped with a PolySep-GFC-P Linear column (Phenomenex, Alcobendas, Spain) and calibrated using pullulan standards (Shodex, Munich, Germany) with molecular weights ranging from 6300 to 642,000 g/mol. Measurements were conducted in duplicate, with 0.1 wt.% carrageenan solutions prepared in 0.1 M NaCl at 40 °C.

### 4.4. Phase States and Rheological Characterization of Gels

Solutions of NaCl and KCl with four different ionic strengths (0.01, 0.1, 0.5, and 1 mol/L) were prepared by dissolving the appropriate amounts of salt in distilled water. Solutions of extracted KI and commercial K+I were prepared with 3 different total carrageenan concentrations (0,5, 1, and 2 wt.%) by dissolving, with continuous magnetic stirring at 80 °C for 1–2 h, the appropriate amounts of carrageenan in 5 mL of salt solution. In the preparation of KI samples, solutions of NaCl and KCl were used with KI treated with NaOH and KOH, respectively, to obtain a single-cation type, Na^+^ or K^+^. After preparation, all flasks were stored at room temperature (25 °C) for 24 h in a stand still position. After that time, flasks were gently tilted to visually inspect the phase state of the carrageenan samples. Samples were thus sorted into solutions (S); stable suspensions (SS); phase-separated (settled) suspensions (PSS); gels (G); and gels with syneresis (GS). Pictures of representative samples are given in Figure S3 in the Supplementary Materials.

Gel viscoelastic properties were measured using a stress-controlled rotational AR-G2 rheometer (TA Instruments Ltd., New Castle, DE, USA), equipped with a 25 mm parallel-plate geometry and a gap of 500 μm. To prevent water evaporation during testing, dodecane was applied around the sample rim. A time sweep was performed, where hot solutions were cooled from 85 °C to 25 °C over 1.5 h to assess the carrageenan sol–gel transition. The strain amplitude and frequency were set to 0.5% and 1.0 Hz, respectively, essentially to avoid any strain-induced change in the gel-setting behavior. During cooling, the rheometer’s gap was adjusted to account for the thermal expansion of the plates, while the normal force was maintained at 0 ± 1 N to compensate for potential volume changes during the sol-to-gel transition. Subsequently, the mechanical spectrum of the hydrogels was measured at 25 °C using a frequency sweep from 100 to 0.01 Hz, with a strain amplitude of 0.5%, essentially to keep the strain within the linear regime of viscoelasticity. Lastly, the linear and large deformation viscoelastic behavior of the hydrogels was analyzed at 25 °C by performing a strain amplitude sweep between 0.1% and 1000%, with a fixed frequency of 1 Hz.

## Figures and Tables

**Table 1:** Chemical compositions (carrageenan diads from the kappa-carrageenan family in mol.%), molecular mass Mw (in 10^5^ g/mol), and polydispersity index PDI of the hybrid carrageenans extracted from commercial seaweeds and alkali modified with KOH or NaOH. The chemical compositions of the commercial kappa-carrageenan (KAPPA) and the commercial iota-carrageenan (IOTA) are also given.

Sample	KOH						Sample	NaOH					
	ν	μ	ι	κ	Mw	PDI		ν	μ	ι	κ	Mw	PDI
B	0	0	96 ± 5	4 ± 2	5.4 ± 0.1	4.1	B′	1 ± 1	4 ± 4	90 ± 2	5 ± 1	6.1 ± 1.5	4.7
C	3 ± 1	0	91 ± 1	6 ± 2	5.8 ± 0.1	1.6	C′	0 ± 5	0	95 ± 2	5 ± 4	8.9 ± 1.2	6.2
E	0	0	44 ± 6	56 ± 4	3.1 ± 0.1	2.1	E′	1 ± 5	0	42 ± 6	57 ± 3	4.4 ± 0.1	2.8
F	5 ± 5	0	47 ± 1	48 ± 1	2.5 ± 0.1	4.6	F′	1 ± 5	0	45 ± 5	54 ± 3	2.9 ± 0.1	2.4
I	0	0	10 ± 5	90 ± 4	9.6 ± 0.3	3.4	I′	0	0	10 ± 5	90 ± 5	7.9 ± 0.3	4.5
J	0	0	13 ± 3	87 ± 5	8.4 ± 0.1	2.5	J′	0	0	12 ± 6	88 ± 4	10.6 ± 0.1	2.6
K	0	0	5 ± 5	95 ± 5	5.8 ± 0.1	2.9	K′	0	0	7 ± 5	93 ± 4	9.9 ± 0.1	2.7
M	0	0	31 ± 2	69 ± 1	6.5 ± 0.1	2.8	M′	0	4 ± 5	29 ± 1	67 ± 3	5.3 ± 0.1	2.9
KAPPA	0	0	0	100 ± 1	11.3 ± 0.5	2.7	D′	2 ± 2	0	88 ± 1	10 ± 6	14.7 ± 0.2	3.9
IOTA	0	0	92 ± 1	8 ± 5	9.0 ± 0.2	4.7	H′	0 ± 5	1 ± 5	37 ± 6	62 ± 1	3.1 ± 0.1	2.6

**Table 2:** Thermorheological properties of gels formed in KCl. Gel transition temperatures (Tg and T2), gel elastic moduli *G0* measured at equilibrium at 25 °C, and large deformation properties (LAOS) quantified by the strain *γ_F_* for onset on gel-to-fluid shear-induced transition and qualified by the strain-hardening (HARD) or strain-softening (SOFT) behavior occurring at a strain smaller than *γ_F_*.

Gelling Conditions	Samples	Tg (°C)	T2 (°C)	*G0* (Pa)	*γ_F_* (%)	LAOS
2 wt.%–0.01 M KCl	C	43.6 ± 0.2	31.7 ± 0.1	2180 ± 17	377 ± 30	SOFT
	10K + 90I	47.6 ± 0.3	34 ± 2	1022 ± 20	118 ± 13	SOFT/HARD
1 wt.%–1 M KCl	C	>85	60 ± 2	48.7 ± 0.3	331 ± 14	SOFT
	10K + 90I	79.4 ± 0.8	46 ± 3	237 ± 2	556 ± 34	SOFT/HARD
2 wt.%–1 M KCl	C	85.4 ± 4.3	74.3 ± 0.5	985 ± 6	404 ± 30	SOFT/HARD
	10K + 90I	>85	-	11,281 ± 238	388 ± 30	SOFT
2 wt.%–0.01 M KCl	E	66.1± 0.2	54 ± 1	7831 ± 23	37 ± 5	SOFT
	60K + 40I	47.5 ± 0.4	39.5 ± 0.5	15,351 ± 46	60 ± 7	SOFT/HARD
1 wt.%–0.5 M KCl	E	>85	-	2391 ± 14	35 ± 4	SOFT
	60K + 40I	>85	-	157 ± 1	104 ± 10	SOFT
2 wt.%–0.5 M KCl	E	>85	-	14,860 ± 134	11 ± 1	SOFT
	60K + 40I	>85	-	357 ± 2	92 ± 4	HARD
0.5 wt.%–0.01 M KCl	K	43.5 ± 1.5 *	-	490 ± 4	123 ± 6	HARD
	90K + 10I	32 ± 1 *	27 ± 1	535 ± 5	136 ± 7	HARD
0.5 wt.%–0.5 M KCl	K	>85	-	257 ± 2	100 ± 9	SOFT
	90K + 10I	>85	-	1650 ± 61	25 ± 2	SOFT
2 wt.%–0.5 M KCl	K	>85	-	20,476 ± 71	18 ± 2	SOFT
	90K + 10I	>85	-	81,921 ± 4012	3.7 ± 0.6	SOFT

*: no crossover between G′ and G″, rather the temperature where a step rise in G′ occurs.

**Table 3:** Thermorheological properties of gels formed in NaCl. Gel transition temperatures (Tg and T2), gel elastic moduli *G0* measured at equilibrium at 25 °C, and large deformation properties (LAOS) quantified by the strain *γ_F_* for onset on gel-to-fluid shear-induced transition and qualified by the strain-hardening (HARD) or strain-softening (SOFT) behavior occurring at a strain smaller than *γ_F_*.

Gelling Conditions	Samples	Tg (°C)	T2 (°C)	*G0* (Pa)	*γ_F_* (%)	LAOS
2 wt.%–0.01 M NaCl	C′	30.76 ± 0.5	29.3 ± 0.1	738 ± 3	181 ± 5	SOFT
	10K + 90I	52.0 ± 1.5	30 ± 1	1400 ± 128	155 ± 13	SOFT/HARD
1 wt.%–1 M NaCl	C′	83.9 ± 3.4	-	100 ± 60	391 ± 47	SOFT
	10K + 90I	55.6 ± 0.3	-	800 ± 400	25 ± 3	SOFT
2 wt.%–1 M NaCl	C′	<25	-	80 ± 20	355 ± 30	SOFT
	10K + 90I	>85	62 ± 3	410 ± 50	315 ± 38	SOFT/HARD
2 wt.%–0.01 M NaCl	K′	39.4 ± 0.4	40.5 ± 0.5	18,226 ± 111	36 ± 5	SOFT
	90K + 10I	44.5 ± 0.5 *	-	3832 ± 140	100 ± 5	HARD
1 wt.%–0.5 M NaCl	K′	53 ± 1 *	-	3384 ± 82	41 ± 2	HARD
	90K + 10I	46.0 ± 0.3 *	-	6797 ± 45	23 ± 1	SOFT
2 wt.%–0.5 M NaCl	K′	50 ± 1 *	-	3118 ± 96	11 ± 4	HARD
	90K + 10I	54.7 ± 0.3 *	-	25,535 ± 311	49 ± 7	HARD

*: no crossover between G′ and G″, rather the temperature where a step rise in G′ occurs.

## Data Availability

The raw data supporting the conclusions of this article will be made available by the authors on request.

## Supplementary Materials

The following supporting information can be downloaded at: https://www.mdpi.com/article/10.3390/gels11030157/s1, Figure S1: Proton NMR spectra of carrageenans extracted from Euchema (samples B and C), Iridaea (samples E and F), Kappaphycus (samples I, J, K) and Mastocarpus (sample M) and modified with KOH; Figure S2: Proton NMR spectra of carrageenans extracted from Euchema (samples B′ and C′), Iridaea (samples E′ and F′), Kappaphycus (samples I′, J′,K′) and Mastocarpus (sample M′) and modified with NaOH; Figure S3: Representative images of the different states of samples. A and B: gel without syneresis (blend 70K+30I, 2 wt.% in 0.01 mKCl); C1, C2: clear gel with water syneresis (blend 90K+10I, 2 wt.% in 0.01 M KCl); D1: turbid gel (sample B, 2 wt.% in 1 M KCl); D2: turbid gels with precipitated particles (sample M, 2 wt.% in 0.5 M KCl); E: turbid gel (sample B, 2 wt.% in 1 M KCl); F: turbid gel with water syneresis (sample M, 2 wt.% in 0.5 M KCL); G: clear solution (blend 50K+50I, 0.5 wt.% in 0.01 M NaCl); H: turbid solution (sample F, 2 wt.% in 0.01 M KCl); I: stable suspension (sample I, 0.5 wt.% in 1 M KCl); J: phase separated (settled) suspension (sample E′, 2 wt.% in 0.5 M NaCl); Figure S4: Temperature dependence of the storage modulus—G′ (full squares) and loss modulus—G″ (open squares) of hybrid carrageenan (KI) samples (in blue) compared to their respective commercial K+I carrageenan blends (in red) at various polymer concentrations and ionic strengths in KCl solutions. Each row represents a different hybrid carrageenan and blend pair, while each column shows results at different concentrations and ionic strengths. Top row—sample C vs. K+I 10%K+90%I at (A) 2 wt.% KI or K+I in 0.01 M KCl; (B) 1 wt.% KI or K+I in 1 M KCl; (C) 2 wt.% KI or K+I in 1 M KCl; middle row—sample E vs. K+I 60%K+40%I at (D) 2 wt.% KI or K+I in 0.01 M KCl; (E) 1 wt.% KI or K+I in 0.5 M KCl; (F) 2 wt.% KI or K+I in 0.5 M KCl; and bottom row—sample K vs. K+I 90%K+10%I at (G) 0.5 wt.% KI or K+I in 0.01 M KCl; (H) 0.5 wt.% KI or K+I in 0.5 M KCl; (I) 2 wt.% KI or K+I in 0.5 M KCl; Figure S5: Temperature dependence of the storage modulus—G′ (full squares) and loss modulus—G″ (open squares) of hybrid carrageenan (KI) samples (in blue) compared to their respective commercial K+I carrageenan blends (in red) at various polymer concentrations and ionic strengths in NaCl solutions. Each row represents a different hybrid carrageenan and blend pair, while each column shows results at different concentrations and ionic strengths. Top row—sample C′ vs. K+I 10%K+90%I at (A) 2 wt.% KI or K+I in 0.01 M KCl; (B) 1 wt.% KI or K+I in 1 M KCl; (C) 2 wt.% KI or K+I in 1 M KCl; and bottom row—sample K′ vs. K+I 90%K+10%I at (D) 2 wt.% KI or K+I in 0.01 M KCl; (E) 1 wt.% KI or K+I in 0.5 M KCl; (F) 2 wt.% KI or K+I in 0.5 M KCl; Figure S6: Mechanical spectra (storage modulus G′, full squares; loss modulus G″, open squares) of hybrid carrageenan (KI) samples (in blue) compared to their respective commercial K+I carrageenan blends (in red) at various polymer concentrations and ionic strengths in KCl solutions. Each row represents a different hybrid carrageenan and blend pair, and each column shows results at different concentrations and ionic strengths. Top row—sample C vs. K+I 10K+90I at (A) 2 wt.% KI or K+I in 0.01 M KCl; (B) 1 wt.% KI or K+I in 1 M KCl; middle row—sample E vs. K+I 60K+40I at (C) 2 wt.% KI or K+I in 0.01 M KCl; (D) 1 wt.% KI or K+I in 0.5 M KCl; and bottom row—sample K vs. K+I 90K+10I at (E) 0.5 wt.% KI or K+I in 0.01 M KCl; (F) 0.5 wt.% KI or K+I in 0.5 M KCl; Figure S7: Mechanical spectra (storage modulus G′, full squares; loss modulus G″, open squares) of hybrid carrageenan (KI) samples (in blue) compared to their respective commercial K+I carrageenan blends (in red) at various polymer concentrations and ionic strengths, in NaCl. Each row represents a different hybrid carrageenan and blend pair: (top) sample C′ vs. K+I 10K+90I, at (A) 2 wt.% in 0.01 M NaCl; (B) 1 wt.% in 1 M NaCl; (C) 2 wt.% in 1 M NaCl; and (bottom) sample K′ vs. K+I 90K+10I, at (D) 2 wt.% in 0.01 M NaCl; (E) 1 wt.% in 0.5 M NaCl; (F) 2 wt.% in 0.5 M NaCl; Figure S8: Large amplitude oscillatory shear tests (storage modulus G′, full squares; loss modulus G″, open squares, as a function of the applied strain) of hybrid carrageenan (KI) samples (in blue) compared to their respective commercial K+I carrageenan blends (in red) at various polymer concentrations and ionic strengths in KCl solutions. Each row represents a different hybrid carrageenan and blend pair, while each column shows results at different concentrations and ionic strengths. Top row—sample C vs. K+I 10K+90I at (A) 2 wt.% KI or K+I in 0.01 M KCl; (B) 1 wt.% KI or K+I in 1 M KCl; (C) 2 wt.% KI or K+I in 1 M KCl; middle row—sample E vs. K+I 60K+40I at (D) 2 wt.% KI or K+I in 0.01 M KCl; (E) 1 wt.% KI or K+I in 0.5 M KCl; (F) 2 wt.% KI or K+I in 0.5 M KCl; and bottom row—sample K vs. K+I 90K+10I at (G) 0.5 wt.% KI or K+I in 0.01 M KCl; (H) 0.5 wt.% KI or K+I in 0.5 M KCl; (I) 2 wt.% KI or K+I in 0.5 M KCl; Figure S9: Large amplitude oscillatory shear tests (storage modulus G′, full squares; loss modulus G″, open squares, as a function of the applied strain) of hybrid carrageenan (KI) samples (in blue) compared to their respective commercial K+I carrageenan blends (in red) at various polymer concentrations and ionic strengths in NaCl solutions. Each row represents a different hybrid carrageenan and blend pair, while each column shows results at different concentrations and ionic strengths. Top row—sample C′ vs. K+I 10K+90I at (A) 2 wt.% KI or K+I in 0.01 M NaCl; (B) 1 wt.% KI or K+I in 1 M NaCl; (C) 2 wt.% KI or K+I in 1 M NaCl; and bottom row—sample K′ vs. K+I 90K+10I at (D) 2 wt.% KI or K+I in 0.01 M NaCl; (E) 1 wt.% KI or K+I in 0.5 M NaCl; (F) 2 wt.% KI or K+I in 0.5 M NaCl.
