# Supplementary material for: Hybrid Carrageenans Versus Kappa–Iota-Carrageenan Blends: A Comparative Study of Hydrogel Elastic Properties
Source: Gels. 2025 Feb 22;11(3):157. doi: 10.3390/gels11030157 (PMC11942092; doi:10.3390/gels11030157)
Supplement: Supplementary file 1 [file gels-11-00157-s001.zip › gels-3481765-supplementary.pdf]

gels

Supplementary Materials

Hybrid Carrageenans Versus Kappa–Iota-Carrageenan Blends: A Comparative Study of Hydrogel Elastic Properties

Maria Alice Freitas Monteiro [1], Bruno Faria [1], Izabel Cristina Freitas Moraes [2] and Loic Hilliou [1,*]

[1] Institute for Polymers and Composites, University of Minho, 5800-048 Guimarães, Portugal; mariaamonteiro13@gmail.com (M.A.F.M.); bruno.faria@dep.uminho.pt (B.F.)
[2] Department of Food Engineering, Faculty of Animal Science and Food Engineering (FZEA), University of São Paulo (USP), Postgraduate Programme in Materials Science and Engineering, 13635-900 Pirassununga, São Paulo, Brazil; bel@usp.br
* Correspondence: loic@dep.uminho.pt

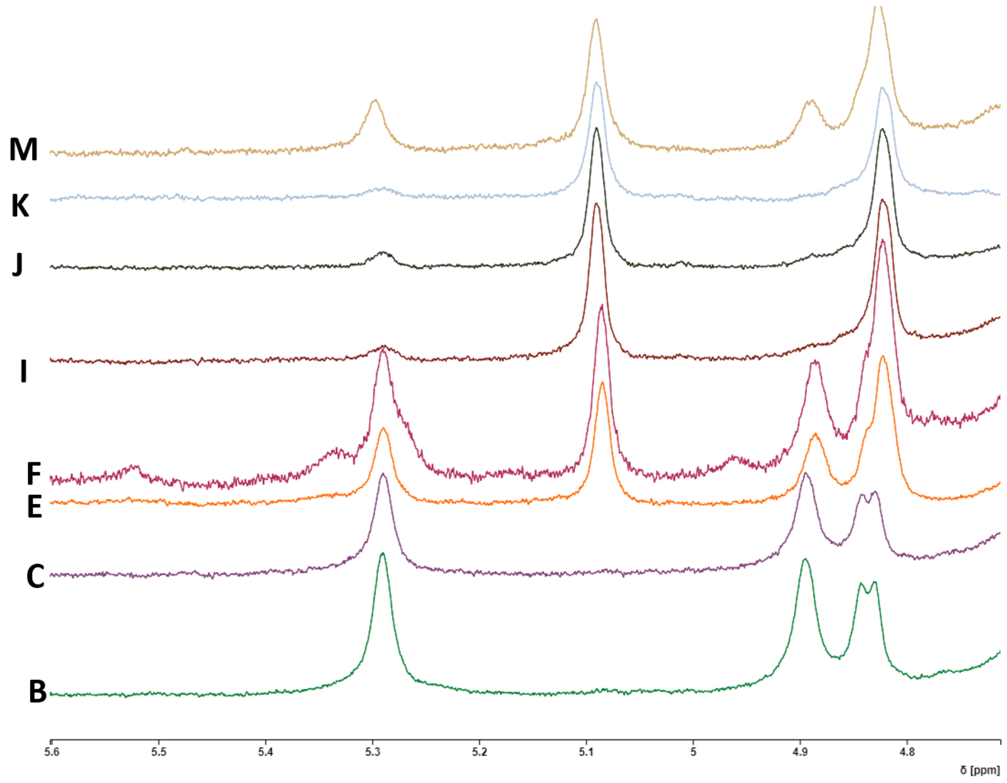

Figure S1. Proton NMR spectra of carrageenans extracted from Euchema (samples B and C), Iridaea (samples E and F), Kappaphycus (samples I,J,K) and Mastocarpus (sample M) and modified with KOH.

Academic Editor: Wei Cui

Received: 31 January 2025
Revised: 18 February 2025
Accepted: 20 February 2025
Published: 22 February 2025

Citation: Monteiro, M.A.F.; Faria, B.; Moraes, I.C.F.; Hilliou, L. Hybrid Carrageenans Versus Kappa–Iota Carrageenan Blends: A Comparative Study of Hydrogel Elastic Properties. *Gels* **2025**, *11*, 157. https://doi.org/10.3390/gels11030157

Copyright: © 2025 by the authors. Licensee MDPI, Basel, Switzerland. This article is an open access article distributed under the terms and conditions of the Creative Commons Attribution (CC BY) license (https://creativecommons.org/licenses/by/4.0/).

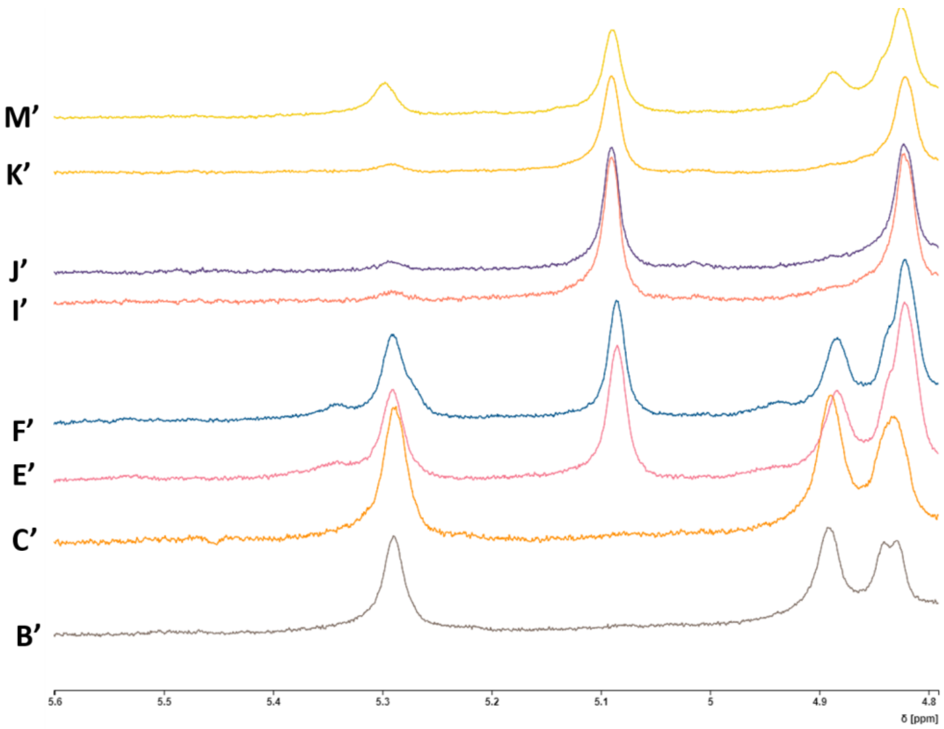

Figure S2. Proton NMR spectra of carrageenans extracted from Euchema (samples B′ and C′), Iridaea (samples E′ and F′), Kappaphycus (samples I′, J′,K′) and Mastocarpus (sample M′) and modified with NaOH.

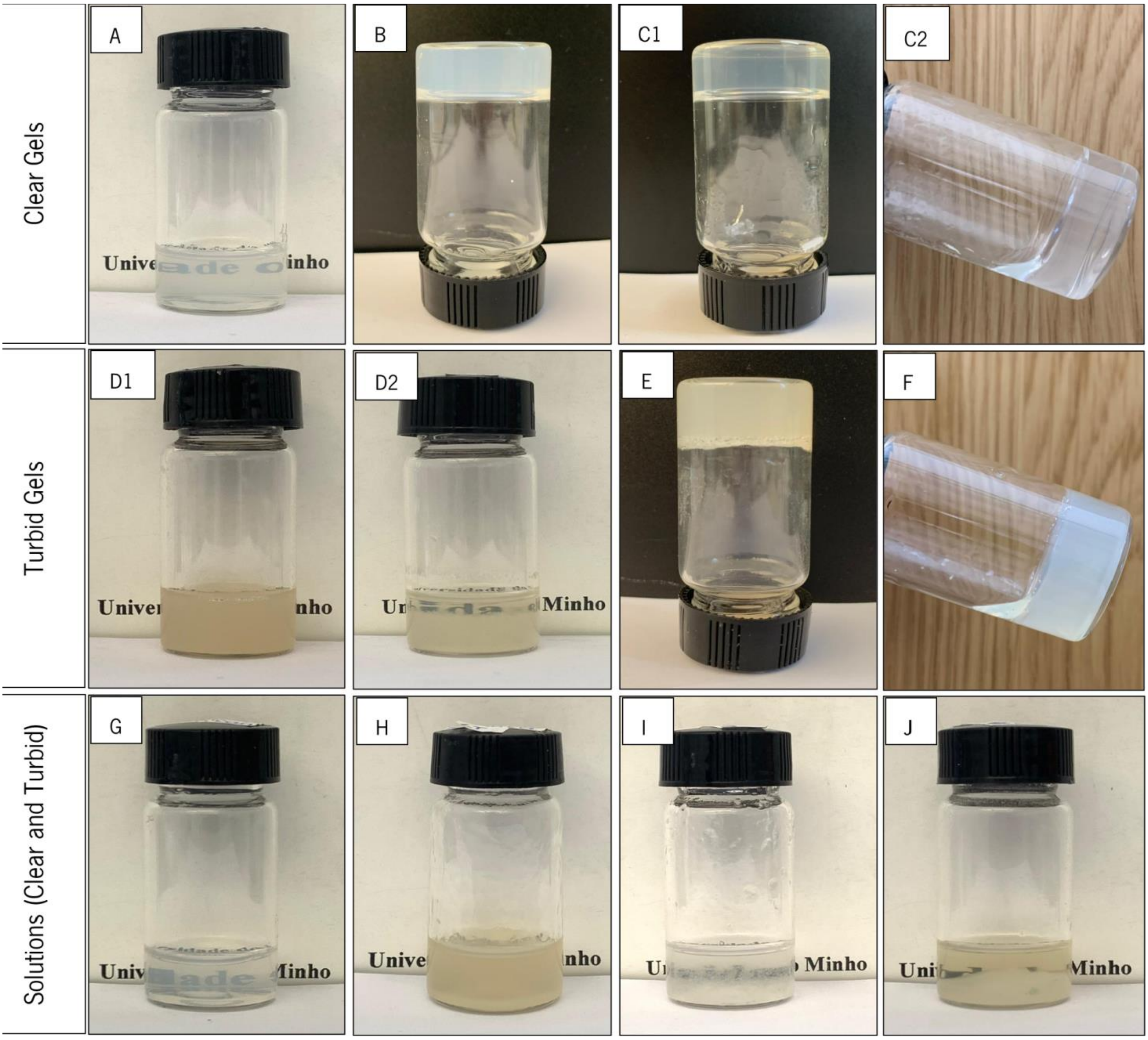

Figure S3. Representative images of the different states of samples. A and B: gel without syneresis (blend 70K + 30I, 2 wt.% in 0.01 mKCl); C1, C2: clear gel with water syneresis (blend 90K + 10I, 2 wt.% in 0.01 M KCl); D1: turbid gel (sample B, 2 wt.% in 1 M KCl); D2: turbid gels with precipitated particles (sample M, 2 wt.% in 0.5 M KCl); E: turbid gel (sample B, 2 wt.% in 1 M KCl); F: turbid gel with water syneresis (sample M, 2 wt.% in 0.5 M KCL); G: clear solution (blend 50K + 50I, 0.5 wt.% in 0.01 M NaCl); H: turbid solution (sample F, 2 wt.% in 0.01 M KCl); I: stable suspension (sample I, 0.5 wt.% in 1 M KCl); J: phase-separated (settled) suspension (sample E', 2 wt.% in 0.5 M NaCl).

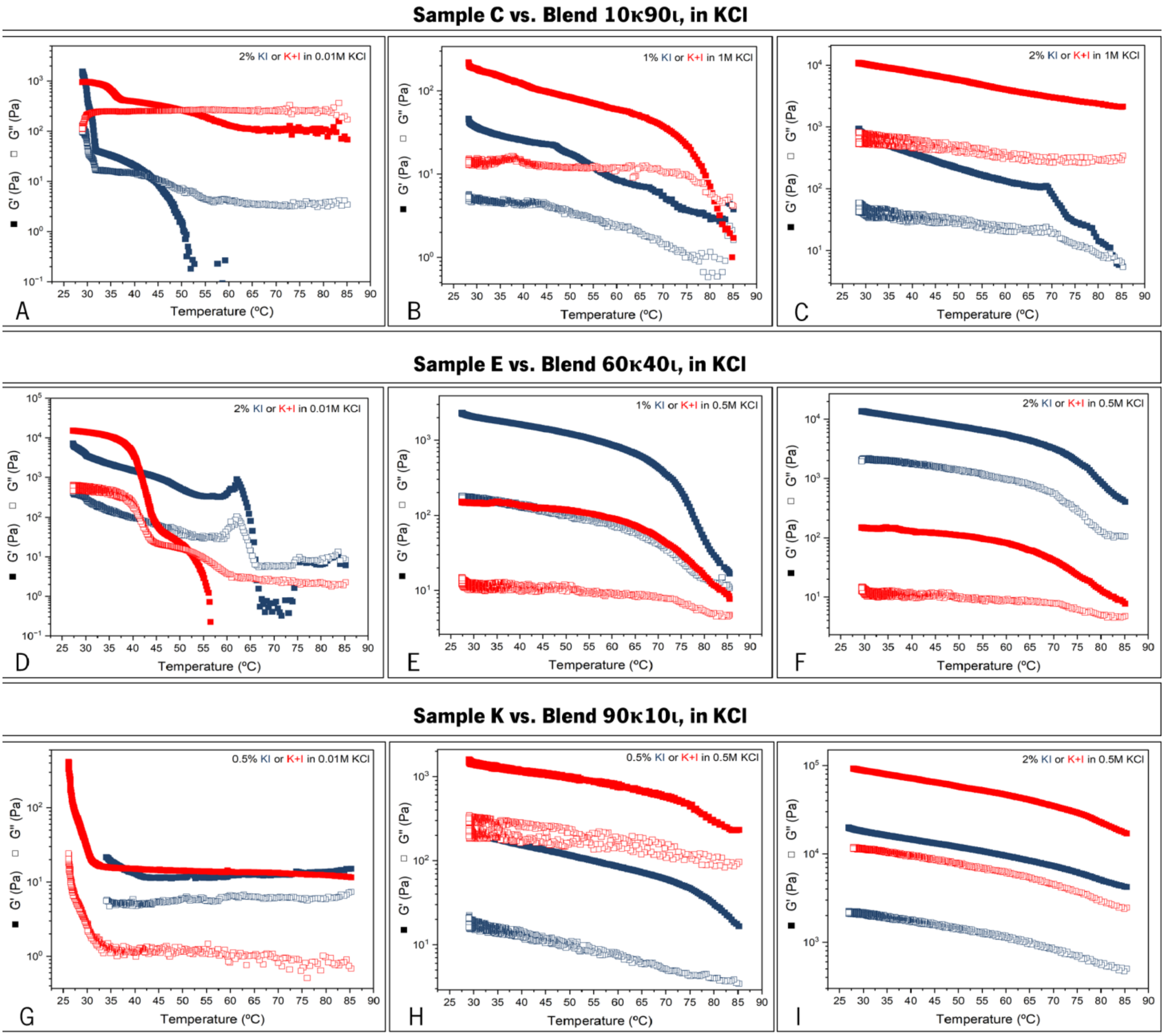

Figure S4. Temperature dependence of the storage modulus - G′ (full squares) and loss modulus - G′′ (open squares) of hybrid carrageenan (KI) samples (in blue) compared to their respective commercial K+I carrageenan blends (in red) at various polymer concentrations and ionic strengths in KCl solutions. Each row represents a different hybrid carrageenan and blend pair, while each column shows results at different concentrations and ionic strengths. Top row - sample C vs. K+I 10%K + 90%I at A) 2 wt.% KI or K+I in 0.01 M KCl; B) 1 wt.% KI or K+I in 1 M KCl; C) 2 wt.% KI or K+I in 1 M KCl; middle row - sample E vs. K+I 60%K+40%I at D) 2 wt.% KI or K+I in 0.01 M KCl; E) 1 wt.% KI or K+I in 0.5 M KCl; F) 2 wt.% KI or K+I in 0.5 M KCl; and bottom row - sample K vs. K+I 90%K+10%I at G) 0.5 wt.% KI or K+I in 0.01 M KCl; H) 0.5 wt.% KI or K+I in 0.5 M KCl; I) 2 wt.% KI or K+I in 0.5 M KCl.

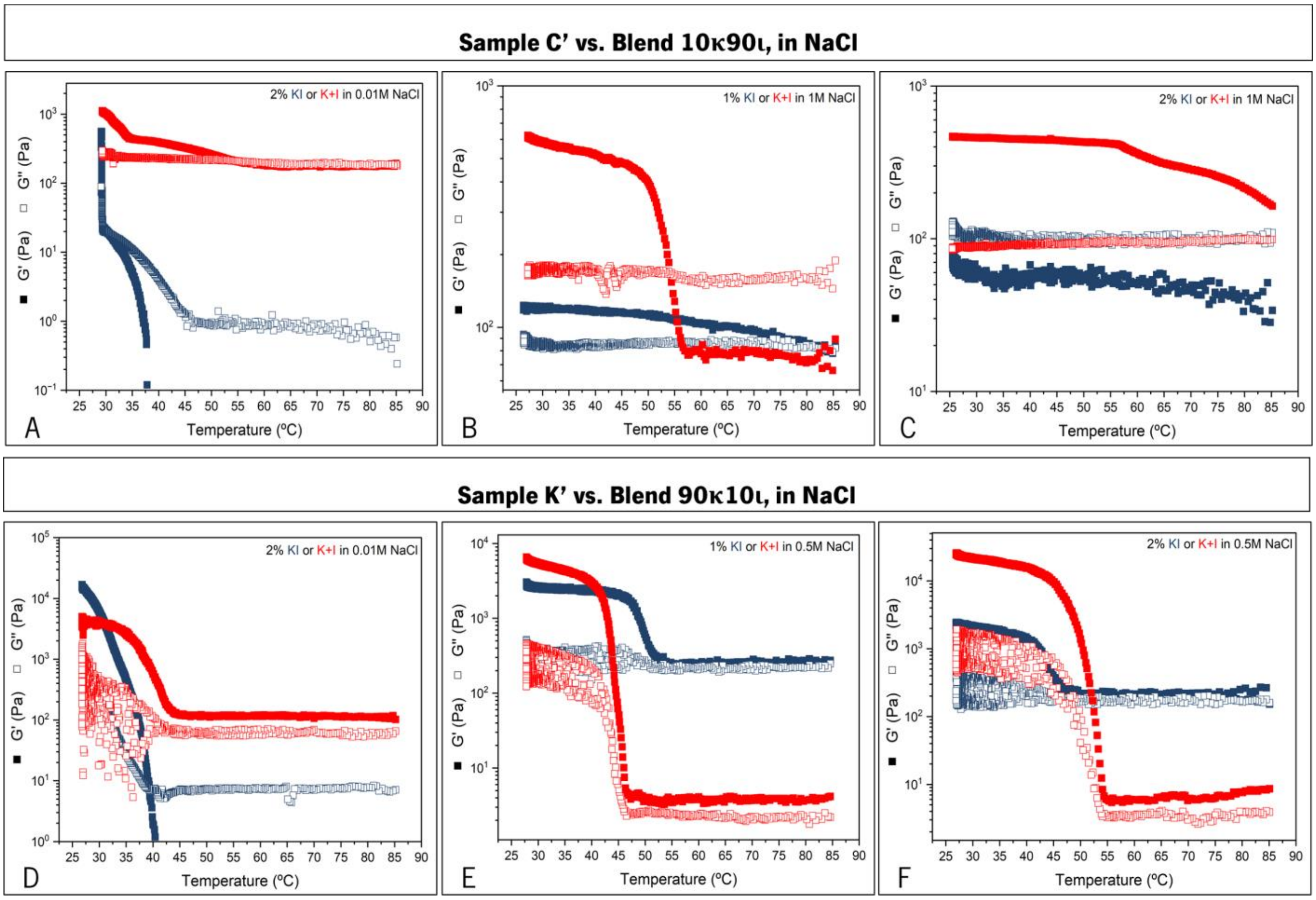

Figure S5. Temperature dependence of the storage modulus - G′ (full squares) and loss modulus - G′′ (open squares) of hybrid carrageenan (KI) samples (in blue) compared to their respective commercial K+I carrageenan blends (in red) at various polymer concentrations and ionic strengths in NaCl solutions. Each row represents a different hybrid carrageenan and blend pair, while each column shows results at different concentrations and ionic strengths. Top row - sample C′ vs. K+I 10%K+90%I at A) 2 wt.% KI or K+I in 0.01 M KCl; B) 1 wt.% KI or K+I in 1 M KCl; C) 2 wt.% KI or K+I in 1 M KCl; and bottom row - sample K′ vs. K+I 90%K+10%I at D) 2 wt.% KI or K+I in 0.01 M KCl; E) 1 wt.% KI or K+I in 0.5 M KCl; F) 2 wt.% KI or K+I in 0.5 M KCl.

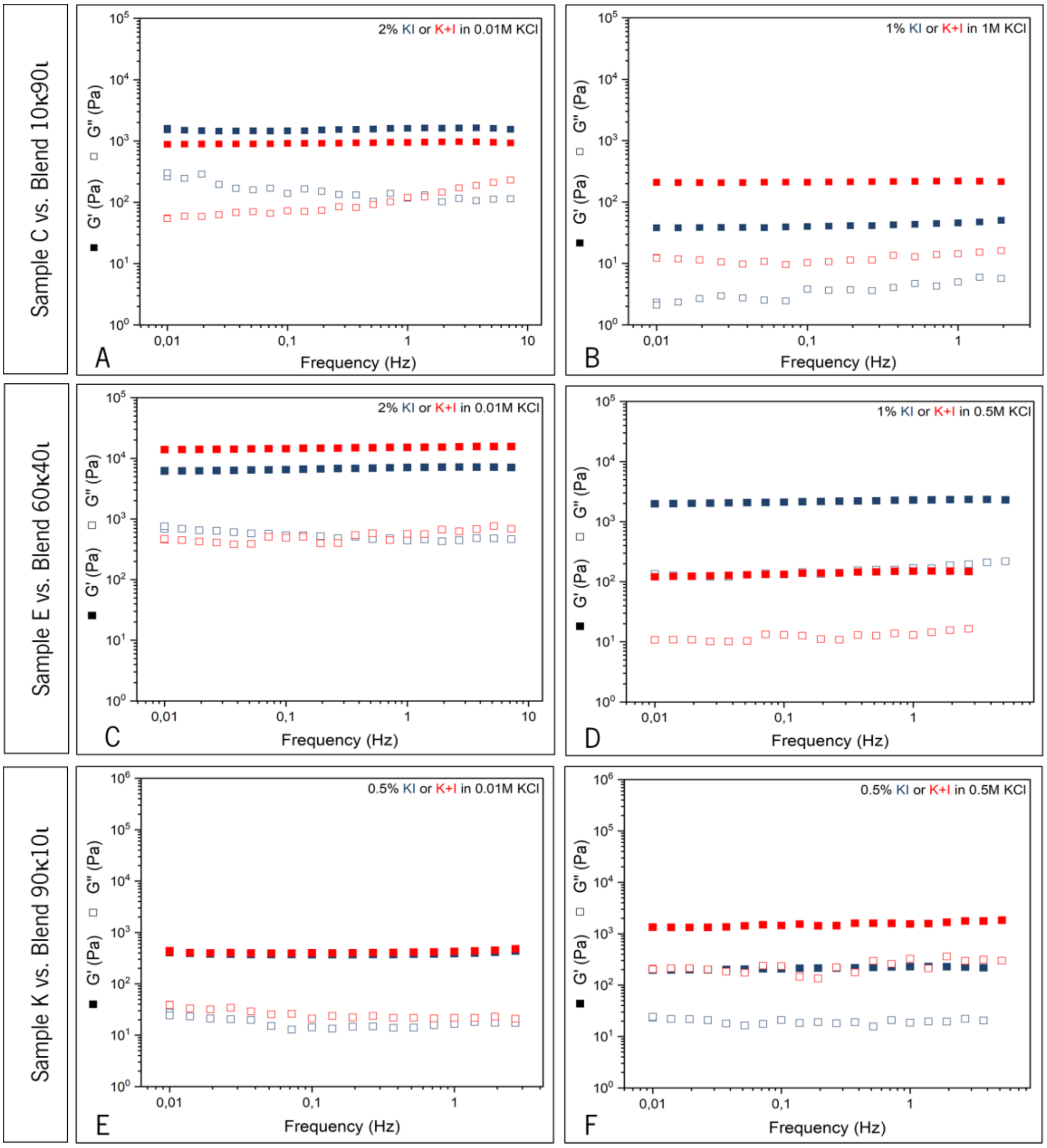

Figure S6. Mechanical spectra (storage modulus G′, full squares; loss modulus G′′, open squares) of hybrid carrageenan (KI) samples (in blue) compared to their respective commercial K+I carrageenan blends (in red) at various polymer concentrations and ionic strengths in KCl solutions. Each row represents a different hybrid carrageenan and blend pair, and each column shows results at different concentrations and ionic strengths. Top row - sample C vs. K+I 10K+90I at A) 2 wt.% KI or K+I in 0.01 M KCl; B) 1 wt.% KI or K+I in 1 M KCl; middle row - sample E vs. K+I 60K+40I at C) 2 wt.% KI or K+I in 0.01 M KCl; D) 1 wt.% KI or K+I in 0.5 M KCl; and bottom row - sample K vs. K+I 90K+10I at E) 0.5 wt.% KI or K+I in 0.01 M KCl; F) 0.5 wt.% KI or K+I in 0.5 M KCl.

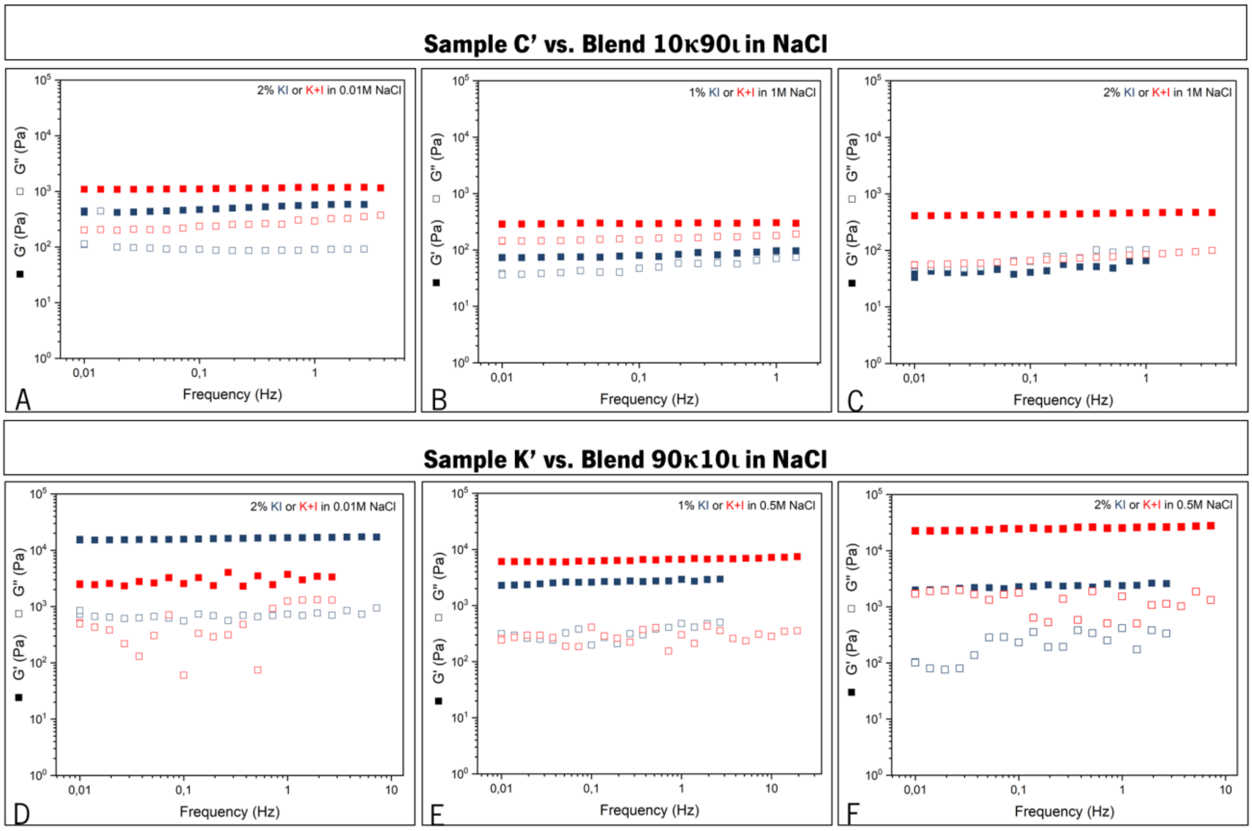

Figure S7. Mechanical spectra (storage modulus G′, full squares; loss modulus G′′, open squares) of hybrid carrageenan (KI) samples (in blue) compared to their respective commercial K+I carrageenan blends (in red) at various polymer concentrations and ionic strengths, in NaCl. Each row represents a different hybrid carrageenan and blend pair: (top) sample C′ vs. K+I 10K+90I, at A) 2 wt.% in 0.01 M NaCl; B) 1 wt.% in 1 M NaCl; C) 2 wt.% in 1 M NaCl; and (bottom) sample K′ vs. K+I 90K+10I, at D) 2 wt.% in 0.01 M NaCl; E) 1 wt.% in 0.5 M NaCl; F) 2 wt.% in 0.5 M NaCl.

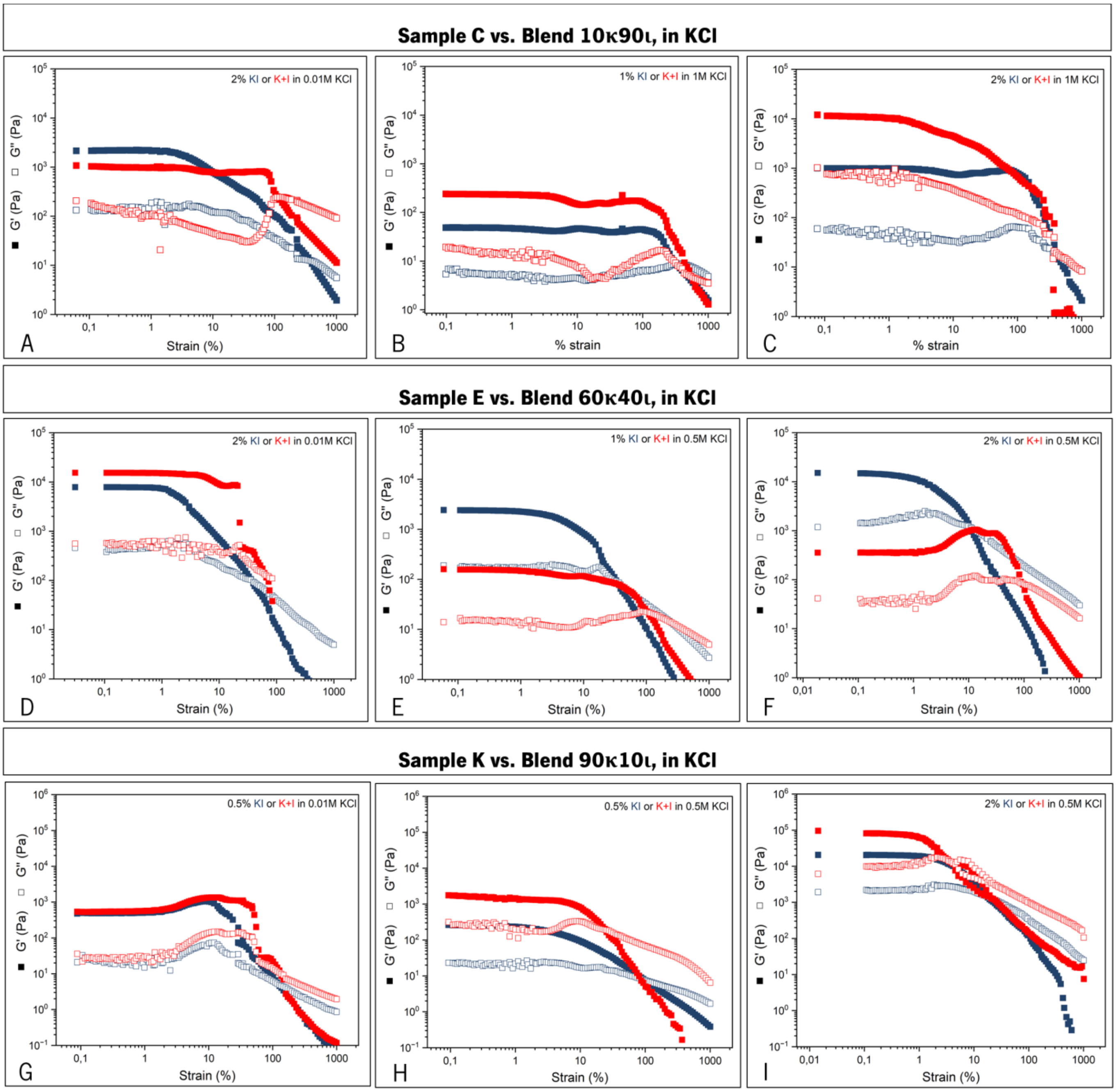

Figure S8. Large amplitude oscillatory shear tests (storage modulus G′, full squares; loss modulus G′′, open squares, as a function of the applied strain) of hybrid carrageenan (KI) samples (in blue) compared to their respective commercial K+I carrageenan blends (in red) at various polymer concentrations and ionic strengths in KCl solutions. Each row represents a different hybrid carrageenan and blend pair, while each column shows results at different concentrations and ionic strengths. Top row - sample C vs. K+I 10K+90I at A) 2 wt.% KI or K+I in 0.01 M KCl; B) 1 wt.% KI or K+I in 1 M KCl; C) 2 wt.% KI or K+I in 1 M KCl; middle row - sample E vs. K+I 60K+40I at D) 2 wt.% KI or K+I in 0.01 M KCl; E) 1 wt.% KI or K+I in 0.5 M KCl; F) 2 wt.% KI or K+I in 0.5 M KCl; and bottom row - sample K vs. K+I 90K+10I at G) 0.5 wt.% KI or K+I in 0.01 M KCl; H) 0.5 wt.% KI or K+I in 0.5 M KCl; I) 2 wt.% KI or K+I in 0.5 M KCl.

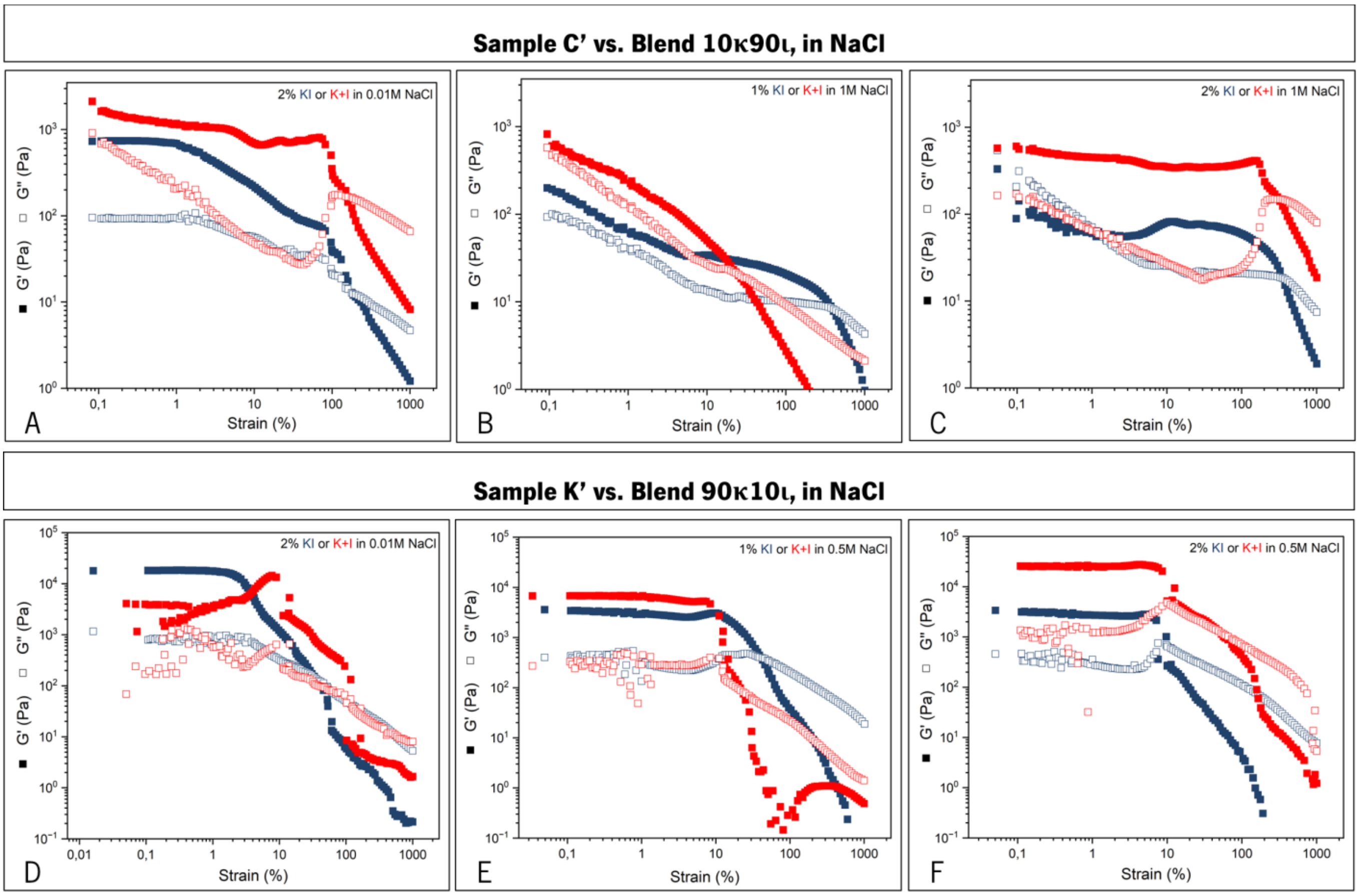

Figure S9. Large amplitude oscillatory shear tests (storage modulus G′, full squares; loss modulus G′′, open squares, as a function of the applied strain) of hybrid carrageenan (KI) samples (in blue) compared to their respective commercial K+I carrageenan blends (in red) at various polymer concentrations and ionic strengths in NaCl solutions. Each row represents a different hybrid carrageenan and blend pair, while each column shows results at different concentrations and ionic strengths. Top row - sample C′ vs. K+I 10K+90I at A) 2 wt.% KI or K+I in 0.01 M NaCl; B) 1 wt.% KI or K+I in 1 M NaCl; C) 2 wt.% KI or K+I in 1 M NaCl; and bottom row - sample K′ vs. K+I 90K+10I at D) 2 wt.% KI or K+I in 0.01 M NaCl; E) 1 wt.% KI or K+I in 0.5 M NaCl; F) 2 wt.% KI or K+I in 0.5 M NaCl.
